# A Case of Renal Cell Carcinoma With Inferior Vena Cava Tumor Thrombus Achieving Pathologic Complete Response Following Combination Therapy With Lenvatinib and Pembrolizumab

**DOI:** 10.1002/iju5.70165

**Published:** 2026-03-15

**Authors:** Hiroki Takayama, Takeshi Sano, Kento Hirao, Yusuke Motoki, Hisanori Taniguchi, Masaaki Yanishi, Katsunori Uchida, Hidefumi Kinoshita

**Affiliations:** ^1^ Department of Urology and Andrology Kansai Medical University Hirakata Japan; ^2^ Department of Urology Takatsuki Red Cross Hospital Takatsuki Japan; ^3^ Department of Pathology and Diagnostics Kansai Medical University Hirakata Japan

**Keywords:** clear cell renal cell carcinoma, immune checkpoint inhibitor, inferior vena cava, pathologic complete response, tyrosine kinase inhibitor

## Abstract

**Introduction:**

The efficacy of neoadjuvant lenvatinib plus pembrolizumab for clear cell renal cell carcinoma with inferior vena cava tumor thrombus remains unclear.

**Case Presentation:**

An 80‐year‐old man was diagnosed with clear cell renal cell carcinoma and tumor thrombus extending into the inferior vena cava. Following neoadjuvant lenvatinib plus pembrolizumab, the primary renal tumor shrank markedly, whereas the inferior vena cava tumor thrombus demonstrated only a slight reduction in size. Robot‐assisted radical nephrectomy and lymphadenectomy were performed; however, conversion to open surgery was required because of an inferior vena cava laceration. Pathological examination revealed no residual viable cancer cells. The patient has remained free of recurrence for 1.5 years postoperatively.

**Conclusion:**

In this case, a pathological complete response was achieved following combination therapy of lenvatinib with pembrolizumab.

## Keynote Message

In a patient with locally advanced RCC involving an IVC tumor thrombus, neoadjuvant lenvatinib plus pembrolizumab was administered to enhance resectability and improve operative safety. IVC injury occurred due to adhesions, representing a drawback of the neoadjuvant therapy; however, a pathological CR was achieved, with no recurrence for 1.5 years postoperatively.

**Abbreviations**
ccRCC: clear cell renal cell carcinoma
CR: complete response
CRP: C‐reactive protein
CT: computed tomography
ICI: immune checkpoint inhibitor
IHC: immunohistochemical
IVC: inferior vena cava
LN: lymph node
MRI: magnetic resonance imaging
PD‐L1: programmed death‐ligand 1
RBC: red blood cell
RCC: renal cell carcinoma
TKI: tyrosine kinase inhibitor
TSH: thyroid‐stimulating hormone

## 1 Introduction

Surgical resection remains the standard curative treatment for nonmetastatic renal cell carcinoma (RCC) [1]. However, in selected patients with large tumors, locally invasive disease, or an inferior vena cava (IVC) tumor thrombus, preoperative systemic therapy may be administered to improve local disease control [2]. Lenvatinib combined with pembrolizumab is a standard regimen for unresectable or metastatic RCC [3]; however, its efficacy as a neoadjuvant therapy has not been fully established. Here, we report a case of clear cell RCC (ccRCC) with IVC tumor thrombus and lymph node (LN) metastasis that achieved a pathological complete response (CR) following lenvatinib plus pembrolizumab.

## 2 Case Presentation

**FIGURE 1:**
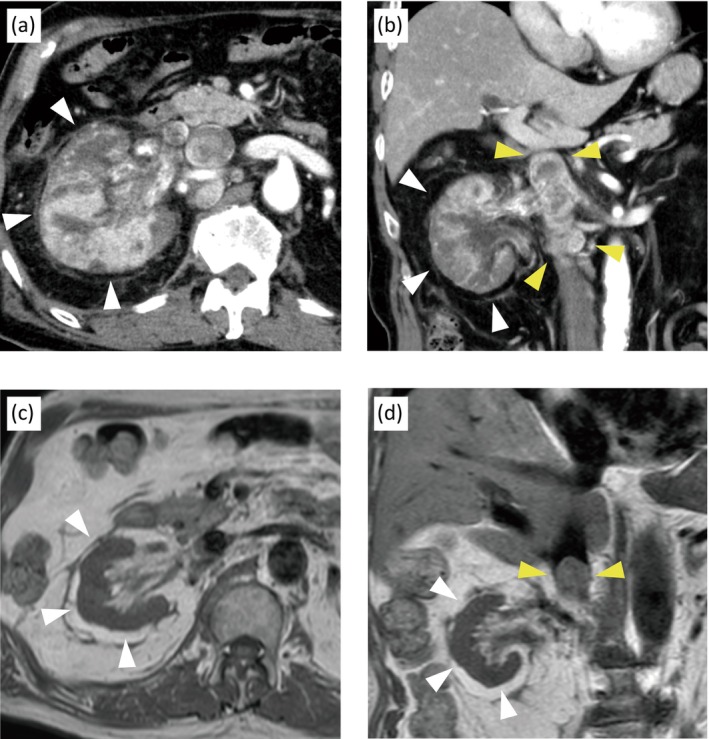
Radiological findings before and after systemic therapy. (a) Axial and (b) coronal contrast‐enhanced computed tomography scans showing a right renal mass with extension into the inferior vena cava (IVC) before systemic treatment. (c) Axial and (d) coronal T_1_‐weighted magnetic resonance imaging obtained after combination therapy of lenvatinib with pembrolizumab. White and yellow arrowheads denote the primary renal tumor and the IVC tumor thrombus, respectively.

An 80‐year‐old man was referred to our hospital for further evaluation of a right renal mass. He had a history of chronic kidney disease. His Eastern Cooperative Oncology Group performance status was 0. Laboratory tests showed elevated creatinine (1.75 mg/dL) and CRP (13.71 mg/dL). His complete blood count, serum calcium, and albumin were all within normal limits. A contrast‐enhanced computed tomography (CT) revealed a large right renal mass measuring approximately 9.0 cm in maximum diameter with heterogeneous enhancement (Figure 1a), para‐aortic LN enlargement measuring 2.0 cm in short‐axis diameter, and tumor thrombus extending into the IVC, consistent with Level II of the Mayo Clinic's thrombus classification (Figure 1b). Histopathologic findings from the percutaneous needle biopsy of the renal mass suggested ccRCC, and the patient was diagnosed with cT3bN1M0 ccRCC.

Systemic therapy with lenvatinib (20 mg) plus pembrolizumab (200 mg) was initiated to achieve local disease control. After four courses, lenvatinib was discontinued due to hypertension and renal function deterioration. Subsequently, pembrolizumab monotherapy was continued through the eighth course. Plain CT and magnetic resonance imaging (MRI) showed that the primary renal tumor had completely disappeared (Figure 1c), whereas the IVC tumor thrombus showed only a slight reduction in size and remained at Level II of the Mayo classification (Figure 1d). The enlarged LN decreased in size to a short‐axis diameter of 0.7 cm.

Subsequently, robot‐assisted nephrectomy was performed using the da Vinci Xi surgical system; however, the procedure was converted to open surgery after an approximately 1.5‐cm longitudinal laceration developed in the lateral wall of the IVC immediately cranial to the renal vein confluence during blunt dissection between the kidney and the IVC, in the setting of severe peritumoral adhesions; the defect was repaired with continuous sutures using 5‐0 Prolene. The surgery was 6 h and 11 min with 1616 mL of blood loss and transfer of 10 units of RBCs. The patient was discharged without postoperative complications, and no perioperative deterioration of renal function was observed. Histopathologically, both the renal tumor and the IVC tumor thrombus showed extensive necrosis and fibrosis, with immune cell infiltration especially in the surrounding tissue. No viable tumor cells were identified in the specimens, suggesting that the ccRCC with IVC tumor thrombus and LN metastasis had achieved a pathological CR to lenvatinib plus pembrolizumab.

**FIGURE 2:**
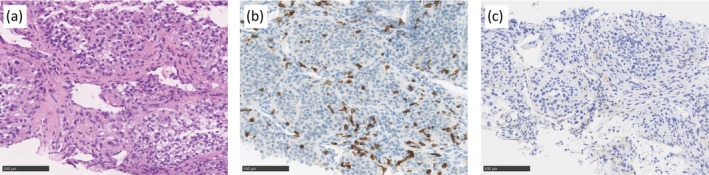
Histopathologic findings in the pretreatment biopsy specimen. Hematoxylin and eosin staining (a), and immunohistochemical staining for CD8 (b) and programmed death‐ligand 1 (c). Scale bars = 100 μm.

**FIGURE 3:**
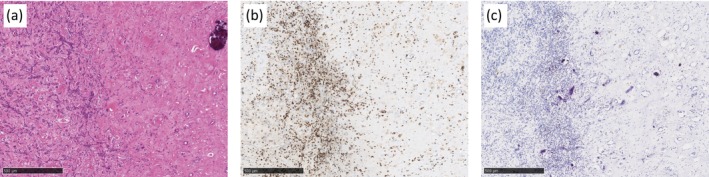
Histopathologic findings in the nephrectomy specimen after combination therapy of lenvatinib with pembrolizumab. Hematoxylin and eosin staining (a) and immunohistochemical staining for CD8 (b) and programmed death‐ligand 1 (c). Scale bars = 250 μm.

Pretreatment, hematoxylin, and eosin staining of the biopsy specimens demonstrated a ccRCC composed predominantly of tumor cells with minimal cytoplasmic clearing, resulting in a more solid, eosinophilic appearance (Figure 2a). Immunohistochemical (IHC) staining of the biopsy specimen demonstrated a marked infiltration of CD8‐positive lymphocytes (Figure 2b) and moderate expression of programmed death‐ligand 1 (PD‐L1) (Figure 2c) within the tumor tissue. Within the necrotic tissue of the nephrectomy specimen obtained after the neoadjuvant therapy (Figure 3a), abundant CD8‐positive lymphocytes were observed (Figure 3b), whereas PD‐L1–positive immune cells remained infrequent (Figure 3c). Postoperatively, combination therapy was not resumed, and the patient has remained free of recurrence for 1.5 years.

## 3 Discussion

Since the combination regimens of immune checkpoint inhibitor (ICI) and tyrosine kinase inhibitor (TKI) have become available as first‐line systemic therapy for unresectable or metastatic RCC, these combination therapies are increasingly being employed as neoadjuvant therapy for patients with locally advanced RCC to improve resectability and surgical safety [4]. Recent meta‐analyses have demonstrated potential benefits of neoadjuvant therapy with ICI–TKI combinations, including improved surgical safety, reduced intraoperative blood loss, and shorter operative times [5].

Several case reports have described the use of neoadjuvant combination therapy for locally advanced RCC, especially with ipilimumab plus nivolumab, pembrolizumab plus axitinib, avelumab plus axitinib, or nivolumab plus cabozantinib [6, 7, 8, 9, 10, 11, 12, 13, 14], and some have achieved pathological CR [7, 8, 9]. Given its high response rate [3], pembrolizumab plus lenvatinib is expected to be a promising neoadjuvant option; however, no studies have attempted this treatment as neoadjuvant therapy for locally advanced RCC. To our knowledge, only two cases of presurgical lenvatinib plus pembrolizumab therapy for metastatic RCC have been reported, both of which demonstrated marked therapeutic responses [15, 16]. These two previous reports and the present case—being the first to apply lenvatinib plus pembrolizumab in the neoadjuvant setting for locally advanced RCC—suggest the potential effectiveness of this regimen as a preoperative therapeutic approach.

A rationale for neoadjuvant lenvatinib plus pembrolizumab was to enhance surgical safety; however, the tumor exhibited remarkable peritumoral adhesion, resulting in laceration to the IVC. Notably, preoperative systemic therapy has been reported to induce peritumoral adhesions [6, 11]. The present case underscores the importance of preoperative communication regarding potential need for conversion to open surgery and advance planning for vascular surgical support when severe adhesions are anticipated. If safe dissection appears challenging, early conversion should be considered to mitigate the risk of vascular injury. Because no established method exists to predict peritumoral or perivascular adhesion, the indication for preoperative systemic therapy should be carefully considered.

In RCC, tumor‐infiltrating lymphocytes have been reported to correlate with a poorer prognosis [17]. Conversely, recent studies have reported that enrichment of CD8‐positive cells predicts poor prognosis, but is associated with favorable responses to ICIs [18]. In the present case, IHC staining of the pretreatment biopsy specimen revealed a high level of CD8‐positive lymphocytes and moderate PD‐L1 expression within the tumor tissue, suggesting that CD8‐dominant antitumor immunity was partially suppressed through the PD‐1/PD‐L1 pathway, which is consistent with a remarkable response to pembrolizumab. Furthermore, IHC staining of the postoperative specimen also demonstrated abundant CD8 expression and negative PD‐L1 expression, suggesting a sustained antitumor immune response even after discontinuation of pembrolizumab or elimination of antigenic burden and the subsequent contraction of the immune response. This patient had locally advanced disease with LN metastasis, indicating a poor prognosis; however, a pathological CR was achieved with a regimen including an ICI. This observation is consistent with the aforementioned research findings [18].

## 4 Conclusion

We used lenvatinib plus pembrolizumab therapy for a patient with a large renal tumor accompanying the IVC tumor thrombus and LN metastasis. Pathologic examination following radical nephrectomy, thrombectomy, and LN dissection confirmed CR, and the patient has remained recurrence‐free for 1.5 years.

## Disclosure

The authors have nothing to report.

## Consent

Informed consent was obtained from the patient.

## Conflicts of Interest

The authors declare no conflicts of interest.

## Data Availability

The data that support the findings of this study are available from the corresponding author upon reasonable request.
